# First person – Sebastian Markert

**DOI:** 10.1242/bio.057760

**Published:** 2020-12-07

## Abstract

First Person is a series of interviews with the first authors of a selection of papers published in Biology Open, helping early-career researchers promote themselves alongside their papers. Sebastian Markert is first author on ‘Overexpression of an ALS-associated FUS mutation in *C. elegans* disrupts NMJ morphology and leads to defective neuromuscular transmission’, published in BiO. Sebastian conducted the research described in this article while a PhD student in Christian Stigloher's lab at Imaging Core Facility, Biocenter, University of Wuerzburg, Germany. He is now a Postdoc in the lab of Shigeki Watanabe in Baltimore, USA, investigating how neurons communicate with each other on the molecular level.


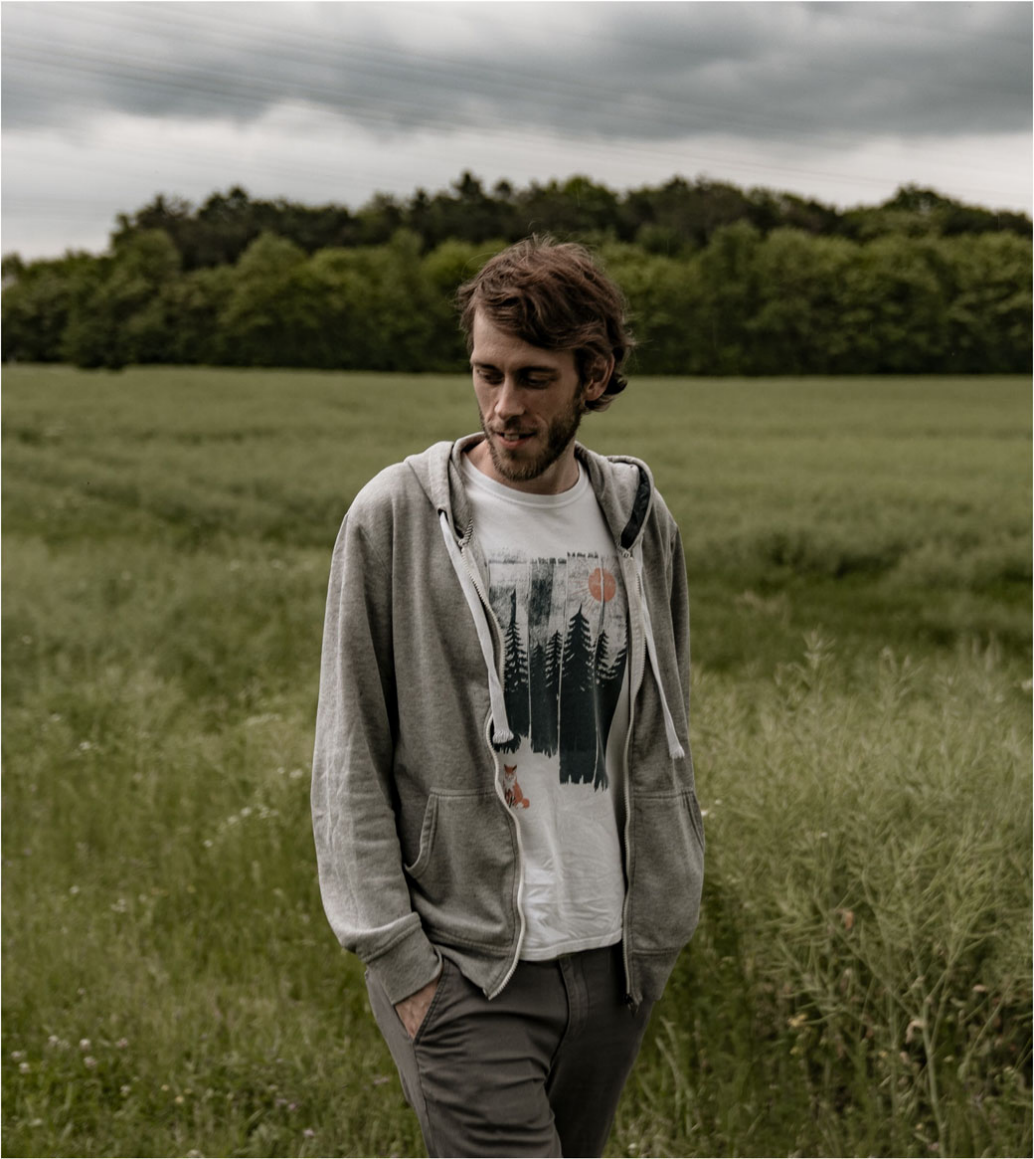


**Sebastian Markert**

**What is your scientific background and the general focus of your lab?**

I am fascinated by almost all fields of biology and luckily had a very broad education at the University of Würzburg in Germany. In the end, neurobiology won, and I joined the Stigloher lab to do my PhD. They use advanced light and electron microscopy techniques for cutting-edge neurobiology research. The main model of the lab is the worm *C. elegans*, that I fell in love with on first sight. It is such a great model organism for basic research.

**How would you explain the main findings of your paper to non-scientific family and friends?**

Muscle wasting diseases are often severe and cause premature death. Scientists are trying to figure out how these diseases work so we can develop treatments. In the one variety we studied, the nerve cells that move the muscle wither and die (and that is causing the wasting of the muscle). Unfortunately, it is very hard to study why these nerve cells die in human patients. Amazingly, though, it is possible to cause the muscle wasting symptoms in tiny worms that are much easier to study. We gave them the mutated human protein that causes the disease and they too died prematurely and could not move very well. We then used light and electron microscopy to look at the nerve cells and also measured their nerve signals with a tiny electrode. We found that the nerve cells have indeed trouble sending their signals to the muscles and they also looked different. This shows that the worm *C. elegans* might be a good model to study the disease in the future and gives some insights as to why and how the nerve cells are dying.

“… *C. elegans* might be a good model to study [ALS] in the future and gives some insights as to why and how the nerve cells are dying.”

**What are the potential implications of these results for your field of research?**

Our study makes a strong case for the usefulness of *C. elegans* as a model for the muscle wasting disease amyotrophic lateral sclerosis (ALS). Future studies with the worm model could offer some decisive breakthroughs in ALS research. We also show how advanced light and electron microscopy techniques can work together to investigate disease mechanisms.

**What has surprised you the most while conducting your research?**

Humans and *C. elegans* worms are vastly different animals. That is why I was so surprised that you can actually just put a mutant human protein that causes a disease into the worm and it develops essentially the same disease. It is amazing how conserved certain biological mechanisms are.

**What changes do you think could improve the professional lives of early-career scientists?**

A career in science is not easy. When I look to myself for strength, I can only try to do science that truly fascinates, inspires, and energizes me. This is what will get me through the challenging parts. I try to have the courage to not spend my time with the kind of science that others want me to do. I hope that early-career scientists get the support and encouragement to do the same.

**Figure:**
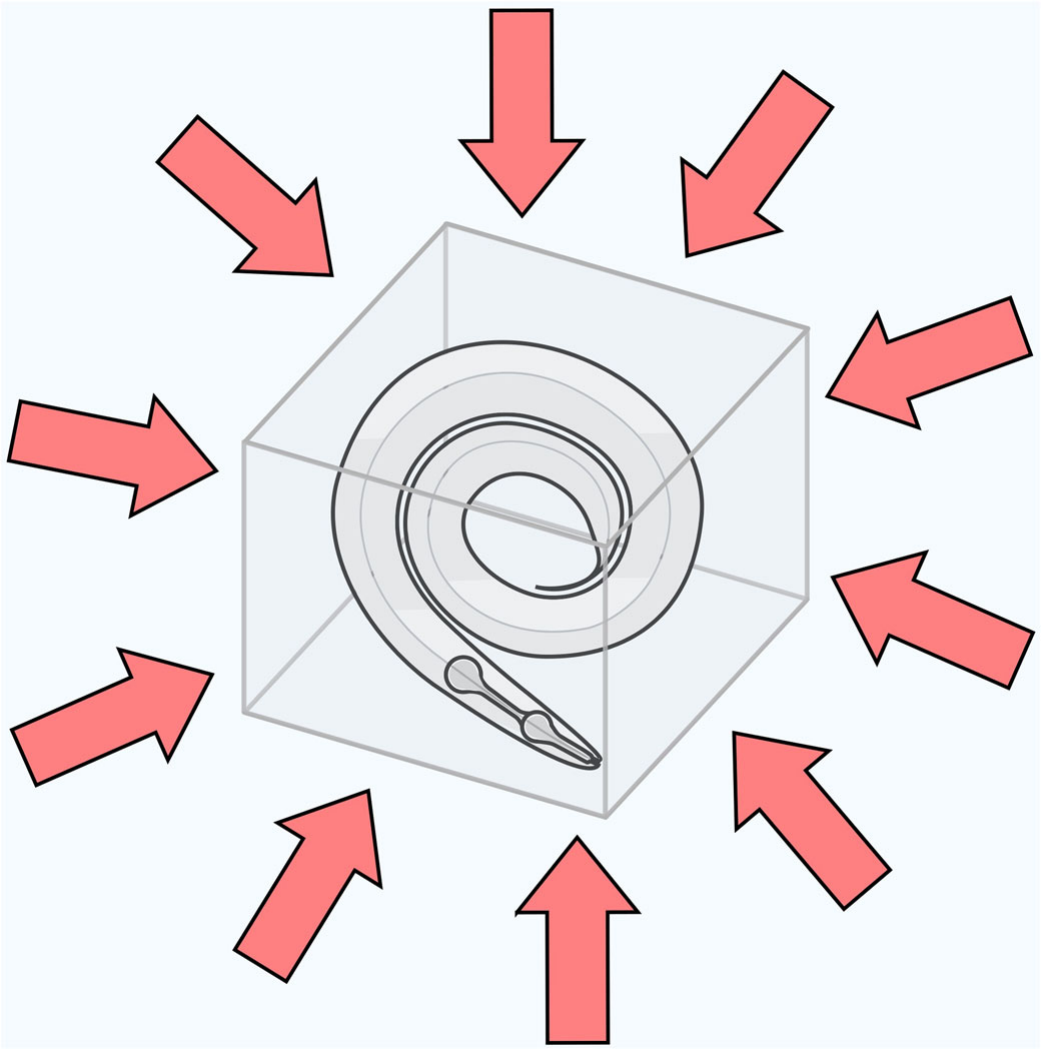
**High-pressure freezing.** To achieve near-native tissue preservation, live worms are cooled so fast that it is like stopping time: proteins and membranes freeze on the spot and are subsequently visualized via electron microscopy.

“When I look to myself for strength, I can only try to do science that truly fascinates, inspires, and energizes me.”

**What's next for you?**

I have graduated and now moved from Germany to the US to do a postdoc at Johns Hopkins University. I am still working with neurons, but this time I study the retina of zebrafish. I want to find out how photoreceptor cells translate light into nerve signals that the brain can understand. These are exciting times and I hope to learn a lot before I try to build my own lab in a few years.
